# Digital Divides in Older People: Assessment of Digital Competencies and Proposals for Meaningful Inclusion

**DOI:** 10.3390/ejihpe15100196

**Published:** 2025-09-26

**Authors:** Rocío Fernández-Piqueras, Rómulo J. González-García, Roberto Sanz-Ponce, Joana Calero-Plaza

**Affiliations:** 1 Faculty of Education and Educational Sciences, Catholic University of Valencia, C/Sagrado Corazón, 5, 46110 Godella, Valencia, Spain; rocio@ucv.es; 2 Campus Capacitas, Catholic University of Valencia, 46110 Godella, Valencia, Spain; rj.gonzalez@ucv.es (R.J.G.-G.); joana.calero@ucv.es (J.C.-P.)

**Keywords:** digital inclusion, older people, digital literacy, digital competence, digital divide, technological literacy

## Abstract

Background: Currently, population aging and the growing incorporation of digital technologies into everyday life highlight the need to ensure the digital inclusion of older adults. This is due to the existence of a significant digital divide that affects this population group, limiting not only their access to services and opportunities but also their emotional well-being and quality of life. The lack of digital skills can generate feelings of exclusion, frustration, and dependence, negatively impacting their mental health and autonomy. Methods: The objective of this study is to assess the level of basic digital competence in 404 older adults using the Scale of Basic Digital Competence in Older Adults (DigCompB_PM) in order to identify existing digital divides and provide empirical evidence for the design of educational interventions that promote the digital inclusion of this population group. To this end, we start with the following research question: Are older adults prepared to face the digital and knowledge society, taking into account personal variables such as age, gender, geographical location, place of residence, and type of cohabitation? Results: The findings reveal that participants scored highest in the dimension related to safety and digital device usage while scoring lowest in online collaboration, indicating a disparity between basic digital skills and collaborative competencies. Cluster analysis further demonstrates that age and previous occupational experience significantly influence digital literacy levels. These results highlight the heterogeneity of digital competence among older adults. Conclusions: The study concludes by emphasising the importance of implementing tailored policies that enhance digital literacy in this population. Key factors such as accessibility, training, and motivation should guide such efforts. Additionally, intergenerational learning emerges as a promising strategy, facilitating the development of digital skills through knowledge exchange and sustained support from younger cohorts.

## 1. Introduction

Population ageing is a global phenomenon that is reshaping our societies. Increasing life expectancy, together with advances in medicine and improved living conditions, have led to a significant increase in the number of older people worldwide. As the United Nations (2017) points out, by 2050, a quarter or more of the world’s population, except in Africa, will belong to this age group. This demographic shift poses new challenges and opportunities and requires an adaptation of our social and political structures. However, it is important to remember that ageing is not just a statistical fact but a biological and social process that implies new demands, relationships and interests, as Urrutia (2018) argues.

In this context, life satisfaction is a multifactorial construct that is influenced by various aspects, from individual characteristics to socio-environmental factors. Numerous studies have shown that life satisfaction is influenced by a wide range of factors, such as educational level, physical or mental health, economic status and psychosocial factors (Papi & Cheraghi, 2021; Phulkerd et al., 2021; Zhang et al., 2022; Banjare et al., 2015). Social support also emerges as a key factor. The presence of a partner or a strong family network is related to higher levels of life satisfaction. According to Chen (2022), geographical context significantly influences levels of well-being, finding significant differences between urban and rural areas and even within apparently homogeneous environments.

In this sense, in the case of older people, digital competence, understood as the ability to effectively use digital technologies to access information, communicate, carry out procedures and participate in the digital society, could emerge as a relevant factor in improving their overall well-being by facilitating access to information, fostering social connection and allowing greater autonomy in daily life. However, the digital divide, or the difficulty this group faces in accessing, using and benefiting from digital technologies compared to younger generations has been associated with inequalities in various aspects of life, such as fear and distrust of lack of digital skills, social isolation, limited access to essential services, communication opportunities or digital citizen participation, among others (Vizconde et al., 2025).

In the ageing process, the transition to retirement, children leaving home and the gradual reduction in interpersonal support networks often lead to substantial changes in the relational sphere of older people. These changes in the social structure increase the likelihood of experiencing adverse emotional states, such as loneliness, depression or anxiety, which are risk factors for overall well-being at this stage of life. The specialised literature has highlighted the need to address these psychosocial dimensions as a fundamental condition for promoting healthy ageing (Colombo & Gónzalez-Gónzalez, 2024). In this context, the potential of digital technologies as tools capable of promoting autonomy, delaying cognitive or functional decline, and contributing to the management of chronic diseases that are highly prevalent in this age group is recognised (Manheim et al., 2019; Peek et al., 2014). However, the health emergency resulting from the COVID-19 pandemic highlighted the heightened vulnerability of the population over 60 years of age, associated with both greater exposure to infection and comorbidities (Banerjee, 2020; Hwang et al., 2020) and an increase in psychosocial problems (isolation, stress, and feelings of loneliness) linked to lockdown and social distancing measures (Conroy et al., 2020).

Moreover, the COVID-19 pandemic has challenged traditional models of ageing, highlighting the importance of social participation and autonomy in older people. The adoption of digital technologies has been presented as a key strategy to promote active and healthy ageing. However, the existing digital divide has limited the access of many older people to technologies, which has had a negative impact on their quality of life and psychosocial well-being (Martínez-Alcalá et al., 2021; Ammar et al., 2021). Social distancing measures implemented during the pandemic have exacerbated this situation by restricting opportunities for social interaction and access to services for this population group. Previous research findings (Martins Van Jaarsveld, 2020; Seifert et al., 2020; Tsamakis et al., 2021) corroborate the relationship between digital inclusion and quality of life. According to Singh and Bajorek (2014), the restrictions faced by older people in the use of digital technologies cannot be understood in isolation but rather as the result of a network of biopsychosocial and sociodemographic factors, including health status, age, lifestyle habits, and gender differences. Therefore, the lack of digital skills in older people significantly reduces their ability to take advantage of the benefits offered by information and communication technologies. This situation limits their autonomy and can have a negative impact on their quality of life by restricting their access to services, information, and opportunities for social interaction.

The insufficient digital literacy of older people, coupled with the accelerating digitisation of society, has led to a situation where many feel forced to adopt technologies that they do not master. This forced adoption, far from being empowering, generates resistance and, in many cases, leads to frustration. As Martínez-Alcalá et al. (2021) and Ammar et al. (2021) point out, this tension between technological demands and the digital capabilities of older people has led to a feeling of exclusion and has hindered their full participation in the digital society. On the other hand, the perspective on ageing has evolved significantly in recent decades, moving from a negative view focused on decline to a more positive perspective that promotes active and fulfilling ageing (Triadó, 2018). In this sense, technologies offer great potential, allowing older people to stay connected, active and participative. However, for digital technologies to be truly beneficial to older people, they need to be adapted to their specific needs and capabilities. Digital inclusion, which involves designing technological solutions that are accessible and easy to use, is essential for them to be able to make the most of the opportunities offered by these technologies (Vizconde et al., 2025).

Therefore, the aim of this study is to assess the digital competence of older people according to the European DigComp 2.2 framework (Vuorikari & Holmes, 2022). Such an assessment is crucial because public policies and programmes delivered by governmental agencies and NGOs frequently rely on frameworks like DigComp to design inclusive digital literacy initiatives. For example, EU policy programmes seek to ensure that older people acquire basic digital skills as part of the Digital Decade goals, and civil society organisations often implement training tailored to seniors’ needs in line with these standards (European Commission, 2023). By examining older adults’ competence via DigComp 2.2, this research can provide evidence to improve the alignment of policy and NGO interventions with actual competence gaps, thereby enhancing digital inclusion in later life.

Based on the above, the following research question arises: Are older people prepared to face the digital and knowledge society, taking into account personal variables such as age and gender, geographical location, place of residence and type of cohabitation? To answer this question, this study assesses the digital competence levels of older people in order to identify the digital divides that impede the digital inclusion of this population group. To achieve this objective, the level of digital competence will be measured in a typical population sample of older people, and the existence of significant differences between the socio-demographic characteristics and the digital level of older people will be analysed.

## 2. Materials and Methods

This is quantitative research with a non-experimental cross-sectional design and a descriptive-comparative scope. The data were collected by means of surveys, using the questionnaire technique, applied to older people in their institutions of reference (day centres or residences) at a single point in time, with the aim of finding out the reality of their level of digital competence and analysing significant differences between groups of elderly people according to their age ranges. The analysis techniques used included descriptive statistics and cluster analysis for the segmented study of the sample, allowing us to examine these differences according to their digital competence and socio-demographic traits.

### 2.1. Participants

This research was carried out in eastern Spain, specifically in the province of Valencia (in its capital and some nearby towns) with people over 65 years of age. Valencia is one of the most populated cities in Spain, with a population of 176,838 older people, whose gender distribution is 40.7% men and 59.3% women, according to data published by the Statistics Office of the Valencia City Council in 2024 (Valencia City Council, 2024). Therefore, based on the sample size calculation for finite populations, with a confidence level of 95% and a margin of error of 5%, a sample of at least 384 subjects is necessary. To obtain the sample, day centres and residences for the elderly in certain neighbourhoods of Valencia and nearby towns were strategically and intentionally selected (rather than randomly). The study was presented to the relevant person in charge of each centre in order to request their voluntary collaboration. As a consequence of this sampling strategy, the sample is biased in terms of socioeconomic level, with a predominance of middle-class profiles. This limitation is mainly due to the geographical location of the participating institutions, which prevents full representation of existing socioeconomic diversity. Nevertheless, efforts were made to include participants with diverse profiles in terms of age, gender, and pre-retirement employment status to increase representativeness in these areas. Finally, there was a non-probabilistic purposive sample of 404 people over 65 years of age, of whom 35.71% were men and 64.29% were women, who freely participated in the study by means of a structured, self-administered survey.

### 2.2. Procedure

To carry out this study, a non-experimental and sectional research design was chosen, supervised and approved by the Ethics Committee of the Catholic University of Valencia (permit number UCV/2021-2022/155). This Committee reviewed and certified the necessary procedures on consent and voluntary participation, information on the purpose of the study, protection of personal data, as well as the guarantee of confidentiality and non-discrimination. Data were collected by means of a paper questionnaire, and an online pass was discarded due to the specific characteristics of the population analysed. Data collection took place between October 2023 and December 2024. To obtain a higher response rate, the sampling points were strategically selected in institutions for the elderly (residences and day centres). In these centres, in small groups and in different shifts, the criteria and purpose of the study were explained beforehand, so that the participants could better understand it and thus complete the questionnaire.

### 2.3. Instrument

The Basic Digital Competence Scale for Older People (DigCompB_PM) (Calero-Plaza et al., 2025) was used to collect the information. This scale is structured into four factors or dimensions and 16 items distributed as follows: F1-Online collaboration through digital devices (6 items), F2-Digital content creation, participation and simple searches (4 items), F3-Basic problem solving and networking training (3 items) and F4-Safety and knowledge of device use (3 items). It measures the digital competence of older adults at a basic level of acquisition of the specific sub-competences that comprise it, in accordance with the Digital Competence Framework for Citizens (Vuorikari & Holmes, 2022). As shown in Calero-Plaza et al. (2025), it has adequate psychometric properties both in terms of internal consistency with Cronbach’s alpha above 0. 70 in the four factors (F1 α = 0.78, F2 α = 0.79, F3 α = 0.76 and F4 α = 0.72), as well as convergent and discriminant validity (with correlations above 0.85 in each of the four dimensions). The instrument has been validated for a specific sample of older people, so the items are numbered consecutively; their descriptions are simple and contain meaningful examples. Finally, there are three possible response options, ‘I don’t know how to do it’, ‘With assistant I can’, ‘I do it with a certain autonomy’, which are appropriate to the context of the participant’s profile. The instrument also includes an initial section to determine the socio-demographic characteristics of the elderly, which will be considered of crucial importance to contrast the digital profiles according to their typical characteristics. This section includes as intervening variables the place of residence (town or city), whether the elderly live alone, accompanied or in a residence, and the professional level of qualification they had before retirement.

### 2.4. Statistical Analysis

In terms of the statistical analyses carried out, an initial descriptive analysis was applied by calculating means, standard deviations, asymmetries, and kurtosis, which allowed an overall characterisation of the variables evaluated. In turn, for the segmentation of the sample, cluster analysis was used using the K-means method (K-means), a consolidated technique for classifying individuals into homogeneous groups according to various variables (Everitt et al., 2011). The selection of the optimal number of clusters was made by evaluating various solutions (between 2 and 5 clusters) based on specific statistical criteria such as the coefficient of determination (R^2^), which reflects the percentage of variance explained by the segmentation obtained; the Akaike information index (AIC) and Bayesian information index (BIC), commonly used to determine the parsimony of the model (Burnham & Anderson, 2004); as well as the Silhouette index, which assesses the quality of separation and internal cohesion of the clusters (Rousseeuw, 1987). The final selection of three clusters was based on the convergence of multiple indicators (R^2^ = 0.64, BIC = 651.88, Silhouette = 0.35, Dunn’s index = 0.07, Calinski-Harabasz index = 356.89). Taken together, these values reflect an adequate balance between model parsimony and classification quality, justifying the three-group solution over other alternatives. Finally, to ensure the robustness of the chosen solution, the Dunn and Calinski-Harabasz indices were additionally calculated to validate the results of the cluster analysis (Dunn, 1974; Calinski & Harabasz, 1974).

In addition, to analyse differences based on categorical variables, a contingency analysis was performed using the chi-squared test (χ^2^), which allows for the detection of associations between nominal or categorical variables (Agresti, 2013). Finally, in order to determine the existence of statistically significant differences between the clusters identified using K-means, a one-way analysis of variance (ANOVA) was carried out, a statistical procedure recommended for comparing means of three or more independent groups (Hair et al., 2014). All statistical analyses performed in this study were carried out using the JASP statistical package (version 0.19.1).

## 3. Results

### 3.1. Characteristics of Participating Users and Levels of Digital Competence According to the Scale of Basic Digital Competence in Older Adults (DigCompB_PM) Administered

The results obtained allow us to identify a general profile of the elderly participant. It is observed that it is a female person, thus fulfilling the general rule that indicates that life expectancy among women is higher than among men (National Institute of Statistics (INE), 2024), with an average age of 75.97 years (SD = 6.83) and 65 years being the minimum age and 99 the maximum. 28.71% reside in the city of Valencia, while 71.28% come from nearby towns; 30% live alone, 56.67% live with a partner or family, and 13.33% live in nursing homes. Lastly, in terms of the level of qualification in the profession they exercised before retirement, 49.04% of the sample had no qualifications at all, compared with the remaining 50.96% who exercised some kind of management position in their respective jobs, whether senior, intermediate or technical.

**Figure 1:**
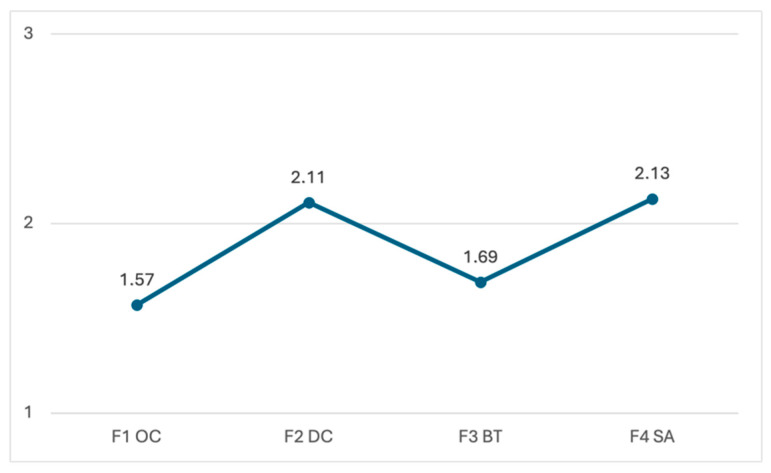
Average level of Digital Competence in Older People according to the Scale of Basic Digital Competence in Older Adults (DigCompB_PM). Note: Axis X: F1 OC: Online collaboration through digital devices; F2 DC: Digital content creation, participation and simple searches; F3 BT: Basic troubleshooting and networking training; F4 SA: Safety and knowledge of use of the devices. Axis Y: 1: I don’t know how to do it 2: With assistance I can 3: I do it with a certain autonomy.

Looking at the descriptive analysis of the results for each of the dimensions assessed, as shown in Figure 1, the dimension with the highest mean score was ‘Safety and knowledge of device use (F4 SA)’, with an mean of M = 2.13 (SD = 0.74) and a reliability index of α = 0.72. Next, the dimension ‘Digital content creation, participation and simple searches (F2 DC)’ had an average rating of M = 2.11 (SD = 0.73) and a reliability index of α = 0.82. Regarding the other dimensions, ‘Basic problem solving and network training (F3 BT)’ had a lower average rating of M = 1.69 (SD = 0.72) and, finally, the dimension with the lowest score was ‘Online collaboration using digital devices (F1 OC)’, with an average rating of M = 1.57 (SD = 0.59).

In terms of distribution, all scales showed moderate skewness (between −0.26 and 0.90) and negative kurtosis (from −1.32 to −0.22). These results indicate that the four dimensions provide consistent and reliable measures of the digital competencies assessed in the sample. These results can be seen in Table 1.

### 3.2. Cluster Analysis

To identify homogeneous clusters in the sample (*n* = 404), a cluster analysis was carried out using the K-means method. The optimal solution corresponded to three clusters, determined using the Bayesian information criterion (BIC) and corroborated by an R^2^ value of 0.64. The Silhouette index obtained (0.35) indicates adequate internal cohesion, although it shows moderate intergroup separation. This value suggests that the cluster structure is statistically acceptable in terms of differentiation between profiles. In conjunction with the parsimony indicators (AIC and BIC) and internal validity (Dunn and Calinski-Harabasz indices), the three-cluster solution can be considered consistent, although the delimitation between groups should be interpreted with caution. The optimal solution corresponded to three clusters, selected on the basis of the Bayesian information criterion (BIC) and supported by a Silhouette index of 0.35, reflecting adequate internal cohesion and moderate external separation. The quality indicators obtained are shown in Table 2.

As can be seen, this solution explains 64% of the total variance (R^2^ = 0.64), indicating that the three identified clusters manage to capture a considerable proportion of the heterogeneity present in the data. Specifically, Cluster 1 (*n* = 95) explains 18% of the heterogeneity, Cluster 2 (*n* = 142) explains 30% and Cluster 3 (*n* = 167) 51%, thus showing a differentiated and significant distribution in the sample analysed. Furthermore, the sum of squares between clusters was 1032.14, out of a total sum of squares of 1612, reflecting an adequate separation. This solution was also supported by the Silhouette index (0.35), together with other indicators such as Dunn’s index (0.07), the Calinski-Harabasz index (356.89), the maximum diameter (4.10) and the minimum separation (0.28), which endorse the statistical quality and validity of the segmentation obtained.

### 3.3. Cluster Profile

Looking at the profile of the clusters, Cluster 1 is characterised by positive scores on all dimensions (F1 = 1.46, F2 = 1.07, F3 = 1.27, F4 = 0.87), suggesting a group with a broader mastery of digital skills, both in online collaboration and in content creation, problem solving and device safety. In contrast, Cluster 2 shows negative or clearly lower scores (F1 = −0.80, F2 = −1.07, F3 = −0.82, F4 = −0.91). This pattern indicates lower proficiency in all four digital dimensions, suggesting lower participation in online collaboration, low initiative in content creation, difficulty in basic problem solving and lower knowledge of digital safety. Finally, Cluster 3 shows intermediate values (F1 = −0.15, F2 = 0.30, F3 = −0.03, F4 = 0.28), suggesting a hybrid profile: they handle certain collaboration and security tools in an acceptable way, although they do not show the same overall strength as Cluster 1, nor do they lag as far behind as Cluster 2.

**Figure 2:**
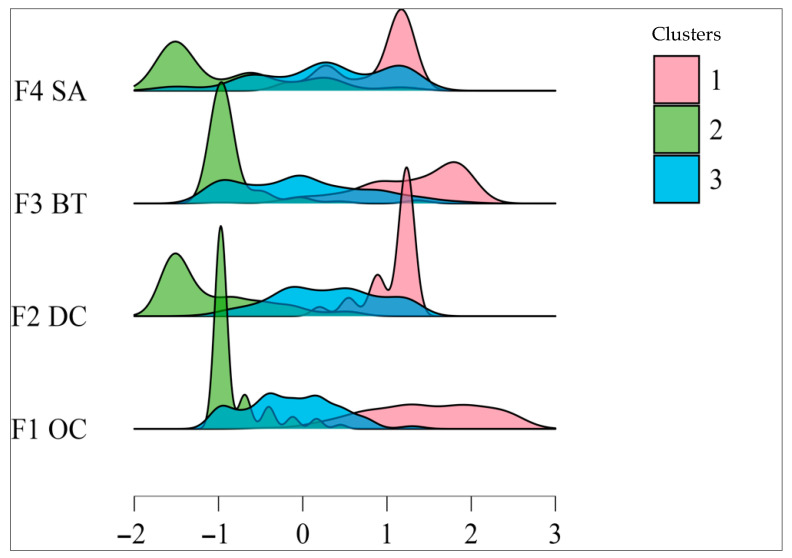
Graph of cluster densities. Note: F1 OC: Online collaboration through digital devices; F2 DC: Digital content creation, participation and simple searches; F3 BT: Basic troubleshooting and networking training; F4 SA: Safety and knowledge of use of the devices.

Figure 2 shows the density graph for each of the clusters (pink: 1, green: 2 and blue: 3), with clearly differentiated distribution patterns in the four dimensions of digital competences: F1 (Online collaboration using digital devices), F2 (Digital content creation, participation and simple search), F3 (Basic problem solving and networking) and F4 (Safety and knowledge of device use). Cluster 1 (pink) is mostly concentrated in positive values, indicating a high and fairly uniform level across all dimensions, while Cluster 2 (green) shows very sharp peaks around negative values, indicating lower digital literacy, and Cluster 3 (blue) shows an intermediate profile with somewhat more dispersed distributions centred around values close to zero. These graphical differences are consistent with the statistical results, as they confirm the existence of three distinct profiles in terms of digital competences within the sample.

### 3.4. Cluster Contrast

To determine whether significant differences exist between the identified clusters, an ANOVA was conducted for each of the four factors assessed. The results (Table 3) showed high levels of significance (*p* < 0.001) in all dimensions, confirming the presence of statistically significant differences between the clusters. This analysis of variance shows that the clusters present differentiated distributions in each factor, reflecting different levels of competence in the dimensions assessed (Table 3). Thus, the segmentation obtained by means of K-means is supported by the statistical results, indicating that the identified clusters have distinctive characteristics consistent with the expected structure.

In terms of socio-demographic variables, the mean age of the participants varies between the groups, with Cluster 1 being the youngest (M = 72.91 years; SD = 5.87), Cluster 2 being the oldest (M = 79.50 years; SD = 7.43) and Cluster 3 being somewhere in between (M = 76.87 years; SD = 6.76). A chi-squared analysis was then calculated to determine the distribution for each of the clusters (Table 4). About the socio-demographic variables analysed, for the variable ‘Sex’,^1^ no statistically significant associations were observed (χ^2^ = 3.96, gl = 2, *p* = 0.14) in the distribution between the groups, as the proportions of men and women were similarly distributed in the three groups. Likewise, neither the variable ‘Do you live’^2^ (χ^2^ = 7.71, gl = 4, *p* = 0.10) nor ‘Locality’^3^ (χ^2^ = 1.13, gl = 2, *p* = 0.57) showed significant associations with cluster membership. These results suggest that differences between clusters do not depend on gender, geographical area or where participants live. In turn, the variable ‘Professional_qualification’^4^ showed a significant association with cluster distribution (χ^2^ = 71.50, gl = 8, *p* < 0.001). It is observed that the lowest categories of professional level are mostly concentrated in Clusters 2 and 3 (48.98% and 41.33%, respectively), while in the highest professional levels there is a higher proportion of individuals belonging to Cluster 1. This result indicates that professional level is related to membership in each cluster, reflecting that those with higher professional training or experience tend to cluster in clusters with better digital competences.

## 4. Discussion

The results obtained in this study reflect a complex reality regarding the digital competence of older people, showing the existence of significant gaps that limit their digital inclusion. In line with previous studies (Charness & Boot, 2022; Rosales & Fernández-Ardèvol, 2020; Vizconde et al., 2025), it is confirmed that the digital literacy of this age group is not homogeneous, with differences depending on various socio-demographic variables. These findings are particularly relevant in a context in which the digitisation of essential services, such as banking, health and public administration, requires older people to have a level of digital competence that they have often not acquired in a formal way.

One of the most relevant findings of this study was the identification of the dimensions with the highest scores on the Digital Competences Scale for Older People (DigCompB_PM) (Calero-Plaza et al., 2025), which were security and knowledge of the use of digital devices (M = 2.13), while the dimension with the lowest score was online collaboration using digital devices (M = 1.57). This result is consistent with previous research that has indicated that older people tend to develop basic skills for interacting with electronic devices but show difficulties in the advanced use of collaborative digital tools and in generating their own content (Gallistl et al., 2020). This situation may be due to a predominantly individual learning model in this age group, where digital interactions tend to focus on family communication and information seeking, rather than collaborative work or content production (Quan-Haase et al., 2018).

In this vein, recent studies have shown that loneliness in old age is associated with independent living, low educational levels, and other specific sociodemographic factors (Pham et al., 2021; Leukel et al., 2023; Zhao et al., 2021). To mitigate these conditions of isolation, mechanisms have been proposed to encourage social participation among older adults by improving their access to information, innovation, and health (Balboni et al., 2011). However, fear of technology can exacerbate perceptions of isolation and limit digital interaction (Conroy et al., 2020). Likewise, barriers associated with the acquisition and use of technology, such as insecurity about data protection, lack of experience, or difficulty in requesting help, directly affect the development of digital competences (Zhao et al., 2021; Hodge et al., 2017; Reis et al., 2021). These limitations have a particularly strong impact on the areas of communication and collaboration in the DigComp framework, where there is a decline in the ability of older people to interact and actively participate in digital environments (Vuorikari & Holmes, 2022).

Despite these limitations, several authors have suggested that social media and digital platforms can become meaningful spaces for interaction that not only strengthen social ties but also contribute to the acquisition of digital skills (Falloon, 2020; Dzokoto et al., 2019; Silva et al., 2020; Tirado-Morueta et al., 2020; Ibarra et al., 2020). This social dimension of the Internet provides additional benefits, such as strengthening critical thinking and developing problem-solving strategies. Likewise, for these benefits to materialise, it is necessary to guarantee the accessibility and usability of devices (Román-García et al., 2016), as well as free and equitable access to the Internet, understood as an indispensable condition for promoting active ageing and full digital participation (Tirado-Morueta et al., 2020; Silva et al., 2020; Kim et al., 2022; Reis et al., 2021; Ibarra et al., 2020; Jin et al., 2019).

Furthermore, the results of the cluster analysis have allowed us to segment the sample into different digital competence profiles, showing that factors such as age and previous work experience significantly influence the level of digital literacy of older people. These findings are consistent with those reported by Van Deursen and Helsper (2018), who show that the lack of early exposure to technology and the absence of structured training are important barriers to the acquisition of digital skills in this population group. This reinforces the idea that digital literacy cannot be considered a spontaneous process in older people but requires specific educational interventions adapted to their learning pace.

A key aspect identified in this study is that older people show difficulties in digital problem solving and network participation, with a score close to the average (M = 1.69) in the corresponding dimension. This finding is relevant because, although some older adults manage to develop basic skills in the use of devices, many others still have difficulties in more advanced digital interaction. This reinforces the idea that lack of confidence in their own technological capabilities is one of the main obstacles to older adults’ participation in complex digital environments (Rosales & Fernández-Ardèvol, 2020). Technological anxiety, understood as the fear of making mistakes or damaging devices, remains a limiting factor preventing full adoption of digital technologies in this group (Czaja et al., 2020).

Furthermore, the analysis of socio-demographic variables has identified that previous educational level and professional qualifications have a significant impact on the development of digital skills in old age. This is also in line with Czaja et al. (2020) who suggest that older people with more professional experience in sectors that have required the use of technology have higher levels of digital competence. This finding reinforces the need to develop differentiated training strategies that not only consider age as the main criterion but also integrate the educational and employment background of the participants.

On the other hand, the lack of accessibility and usability of digital tools remains a fundamental barrier to the technological inclusion of older people. This study confirms that many technological interfaces are not designed with the sensory and cognitive abilities of this age group in mind. Difficulty in using tactile devices, the absence of intuitive interfaces and the complexity of some digital processes have been identified as recurrent issues. Previous research has highlighted the need to develop more inclusive technologies, with an older user-centred design and customisation options adapted to their specific needs (Seifert & Rössel, 2022).

## 5. Conclusions

This research reaffirms the need for public policies that promote digital literacy in older people from an inclusive perspective and adapt to their particularities. Although there are institutional efforts to reduce the digital divide in this age group, the data obtained show that there are still significant challenges in terms of accessibility, training and motivation to use digital tools.

From a social perspective, the results obtained reinforce the idea that the digital literacy of older people should not only be understood as a question of access but also of active participation in the digital society. For these benefits to be effectively achieved, continuous support in the digital learning process is necessary. In this sense, the implementation of intergenerational training programmes can be an effective strategy for the development of digital skills in older people by facilitating learning through the exchange of knowledge with younger generations.

However, this study has some limitations that need to be considered. Firstly, the sample was selected using a non-probabilistic intentional procedure which, although adequate for statistical analysis, limits the possibility of generalising the findings to the entire elderly population. Consequently, the results should be interpreted with caution, as the size and composition of the sample may not reflect the socioeconomic and cultural diversity of this group in other contexts. Furthermore, as this is a cross-sectional study, it is not possible to analyse the evolution of digital competence over time. Secondly, the Silhouette index value reflects acceptable internal cohesion but only moderate separation between groups, which limits the clarity of the segmentation obtained. Finally, the self-administered nature of the questionnaire may have introduced bias in the responses, as some participants may have required support to understand or complete the items. Another limitation to consider is that the information was obtained through self-report questionnaires. Although the scale used has adequate validity and reliability indicators, this method of data collection may be conditioned by the subjective perceptions of the participants or by social desirability biases, which could partially affect the accuracy and objectivity of the responses. Taken together, these limitations reinforce the need to interpret the results with caution and to validate the findings through future studies with larger samples and longitudinal approaches. As for future lines of research, longitudinal studies are suggested to analyse the sustained impact of digital literacy programmes, as well as to explore in greater depth the role of emotional and motivational factors in the digital learning of older people. It would also be valuable to investigate the effectiveness of specific pedagogical methodologies, such as intergenerational learning or the use of adapted accessible technologies, in different geographical and social contexts.

In conclusion, this research contributes to the knowledge on digital competence in the elderly, highlighting the need for specific and contextualised training strategies. The evidence obtained allows for the design of more effective and personalised training programmes, according to the resulting specific groups or clusters, thus promoting greater digital autonomy in the elderly and favouring their integration in an increasingly digitalised society.

## Figures and Tables

**Table 1:** Descriptive and reliability statistics for digital competence dimensions.

	Mean	SD	Skewness	Kurtosis
**F1 OC: Online collaboration through digital devices (α Cronbach = 0.82)**				
OC1—I can collaborate online with other people (e.g., with Google Drive, Dropbox…).	1.40	0.73	1.48	0.51
OC2—I am aware of the data privacy policy of Internet programs (e.g., WhatsApp).	1.58	0.85	0.91	−0.99
OC3—I know how to participate in online citizen surveys (e.g., via WhatsApp, email, social networks…).	1.58	0.79	0.89	−0.83
OC4—I know how to detect inappropriate behavior in the use of cell phones or other devices.	1.72	0.87	0.57	−1.43
OC5—I know how to add text and tags to other people’s videos or photos with my cell phone.	1.64	0.84	0.75	−1.17
OC6—I know how to search for (Internet) images without copyright (without CopyRight).	1.49	0.78	1.16	−0.35
Total	1.57	0.59	0.90	−0.22
**F2 DC: Digital content creation, participation and simple searches (α Cronbach = 0.84)**				
DC1—I know how to send photos, videos or messages over the Internet, e.g., WhatsApp messages).	2.28	0.86	−0.57	−1.42
DC2—I can participate in Internet groups, video calls, networks… (e.g., through WhatsApp).	2.17	0.87	−0.34	−1.61
DC3—I know how to detect if the information that comes to me through the Internet (e.g., WhatsApp, Google, Mail…), is true or not.	1.87	0.90	0.26	−1.72
DC4—I know how to search for information on the Internet (e.g., through Google).	2.10	0.88	−0.19	−1.68
Total	2.11	0.73	−0.26	−1.32
**F3 BT: Basic troubleshooting and networking training (α Cronbach = 0.76)**				
BT1—I know how to change font sizes, change language, adapt screens, …	1.64	0.84	0.76	−1.15
BT2—I know how to solve simple technical problems (e.g., connect wifi, change battery…).	1.68	0.86	0.66	−1.33
BT3—I learn with the Internet (e.g., watching video tutorials, reading google pages, …).	1.75	0.91	0.50	−1.61
Total	1.69	0.72	0.58	−1.09
**F4 SA: Safety and knowledge of use of the devices (α Cronbach = 0.72)**				
SA1—I know that cell phone addiction can cause physical and psychological damage.	2.37	0.89	−0.81	−1.25
SA2—I am aware of the environmental impacts of cell phone use (e.g., carbon emissions, pollution from telecommunication towers…).	1.92	0.96	0.16	−1.90
SA3—I know what personal data I can and cannot send.	2.09	0.93	−0.18	−1.83
Total	2.13	0.74	−0.31	−1.28

**Table 2:** Quality indicators of the cluster model.

Clusters	*n*	R^2^	AIC	BIC	Silhouette
3	404	0.64	603.86	651.88	0.35

Note. R^2^ = variance; AIC = Akaike’s information criterion; BIC = Bayesian information criterion; Silhouette = silhouette index.

**Table 3:** Comparison of means in the dimensions of digital competences between clusters.

Factor	Cluster	Mean	DE	F	*p*
F1 OC: Online collaboration through digital devices	1	2.42	0.40	469.88	<0.001
	2	1.10	0.19		
	3	1.48	0.32		
F2 DC: Digital content creation, participation and simple searches	1	2.88	0.21	707.68	<0.001
	2	1.33	0.42		
	3	2.33	0.44		
F3 BT: Basic troubleshooting and networking training	1	2.60	0.45	419.79	<0.001
	2	1.11	0.30		
	3	1.67	0.54		
F4 SA: Safety and knowledge of use of the devices	1	2.78	0.33	244.81	<0.001
	2	1.46	0.58		
	3	2.33	0.57		

**Table 4:** Distribution of clusters according to sociodemographic variables.

Variable	Cluster			Χ^2^ (gl)	*p*
Sex					
1—Male	29.41%	32.35%	38.24%	3.96 (2)	0.14
2—Female	20.52%	36.57%	42.91%		
Do you live					
1—Alone	16.96%	38.39%	44.64%	7.71 (4)	0.10
2—Accompanied	27.92%	31.67%	40.42%		
3—Residence	17.31%	44.23%	38.46%		
Locality					
1—Town	22.11%	35.44%	42.46%	1.13 (2)	0.57
2—City	26.89%	34.45%	38.66%		
Professional qualification					
1—Senior management	42.11%	21.05%	36.84%	71.50 (8)	<0.001
2—Middle management	38.98%	18.64%	42.37%		
3—Senior technician	53.19%	12.77%	34.04%		
4—Middle technician	18.75%	32.81%	48.44%		
5—Unqualified	9.69%	48.98%	41.33%		

Note. Χ^2^ = chi-squared; gl = degrees of freedom.

## Data Availability

Data from this research may be requested from the authors.

## Notes

1 Sex: Male|Female|Prefer not to say.

2 Do you live: Alone|Accompanied or with family members|In residence.

3 Locality: Town|City.

4 Professional qualification: Senior management|Middle management|Senior technician|Middle technician|Unqualified.
